# Antibiofilm Activities of Biogenic Silver Nanoparticles Against *Candida albicans*

**DOI:** 10.3389/fmicb.2021.741493

**Published:** 2022-01-07

**Authors:** Irshad Ahamad, Fareha Bano, Razique Anwer, Pooja Srivastava, Raj Kumar, Tasneem Fatma

**Affiliations:** ^1^Cyanobacterial Biotechnology Lab, Department of Biosciences, Jamia Millia Islamia, New Delhi, India; ^2^Department of Biology, College of Science and Arts, Taibah University (Female Branch), AlUla, Saudi Arabia; ^3^Department of Pathology, College of Medicine, Imam Mohammad Ibn Saud Islamic University (IMSIU), Riyadh, Saudi Arabia; ^4^Institute of Nuclear Medicine & Allied Sciences, Defence Research & Development Organisation (DRDO), Government of India, New Delhi, India

**Keywords:** biofilm, *Candida albicans*, *Anabaena variabilis*, silver nanoparticles, flow cytometry

## Abstract

Biofilms are microbial colonies that are encased in an organic polymeric matrix and are resistant to antimicrobial treatments. Biofilms can adhere to both biotic and abiotic surfaces, allowing them to colonize medical equipment such as urinary and intravenous catheters, mechanical heart valves, endotracheal tubes, and prosthetic joints. *Candida albicans* biofilm is the major etiological cause of the pathogenesis of candidiasis in which its unobstructed growth occurs in the oral cavity; trachea, and catheters that progress to systemic infections in the worst scenarios. There is an urgent need to discover novel biofilm preventive and curative agents. In the present investigation, an effort is made to observe the role of cyanobacteria-derived AgNPs as a new antibiofilm agent with special reference to candidiasis. AgNPs synthesized through the green route using *Anabaena variabilis* cell extract were characterized by UV–visible spectroscopy. The nanoparticles were spherical in shape with 11–15 nm size and were monodispersed. The minimum inhibitory concentration (MIC) of AgNPs was obtained at 12.5 μg/mL against *C. albicans*. AgNPs 25 μg/mL showed 79% fungal cell membrane permeability and 22.2% ROS production. AgNPs (25 μg/mL) also facilitated 62.5% of biofilm inhibition and degradation. Therefore, AgNPs could be considered as a promising antifungal agent to control biofilm produced by *C. albicans*.

## Introduction

A biofilm is a microbial population or community that lives in organized structures at the surface of a liquid. Biofilm infections do not respond well to available treatment strategies as they evade innate and or/adaptive immune defenses, avoid antimicrobial treatment (Roilides et al., 2015; Vestby et al., 2020). Because of the rise in infections linked to indwelling microorganism on medical devices such as urine and intravenous catheters, mechanical heart valves, endotracheal tubes, and prosthetic joints, microbial biofilms have acquired significance (Nesse et al., 2015). According to the National Institute of Health (NIH), pathogenic biofilms account for more than 80% of all microbial infections (Lohse et al., 2018) of which 75% cases are of vaginal yeast infection in women once or more than once in their lifetime (Perlroth et al., 2007; Yapar, 2014). Around 50% of systemic candidiasis adults patients and approximately 30% of the young population die due to candidiasis correlated with biofilms. Moreover, there is an estimation of 100 thousand deaths because of the infection initiated by biofilm formation (Atriwal et al., 2021).

*Candida albicans* is a major cause of morbidity and mortality in bloodstream infections with indwelling medical devices (IMD) associated infections (Jarvis, 1995; Pappas et al., 2018). Its yeast-to-hypha shape conversion also promotes the formation of a biofilm on inert (medical devices like catheters, shunts, and stents) or biological (skin or mucosa) surfaces, making it more harmful (Kojic and Darouiche, 2004). Biomedical devices inserted during transplantation catheter are the favorable ones as they provide nutrition like glucose from the excreted products (Alim et al., 2018).

The limited number and moderate or low efficacy of antifungal drugs, as well as the severe side effects associated with their administration and the emergence of resistant strains, *Candida* forced us to develop highly effective antifungal drugs with broad-spectrum activity. Mostly all persistent *Candida* infections can be successfully treated with standard fungicides like fluconazole, Amphotericin-B, and echinocandins; however, biofilm-related infections are hard to control by standard treatments (Pappas et al., 2009). *Candida* biofilms are more resistant to azoles than planktonic cells up to 1000 times (Kuhn et al., 2002). The activity of the enzyme 1,3-glucan synthase appears to vary across *Candida* spp. Echinocandins are a class of antifungal medications that block the synthesis of glucan in the fungal cell wall by non-competitive inhibition of the enzyme 1,3 beta-glucan synthase (Kuhn et al., 2002; Choi et al., 2007).

Nanomedicines are getting attention to formulate antibiofilm agents. Our earlier finding related to the antifungal activity of cyanobacteria-derived silver nanoparticles (AgNPs) against *C. albicans* prompted us to understand the role of AgNPs in biofilm inhibition/eradication. An effort was also made to understand its mode of action specially with reference to membrane permeabilization, reactive oxygen species generation and cell cycle through flow cytometer, cell surface hydrophobicity reduction, yeast-to-hypha transition decrease, and cell surface morphology alteration.

## Materials and Methods

### Chemicals Reagents and Microbial Cultures

Dichloro-fluorescein diacetate (DCFH-DA), 4-6-diamidino-2-phenylindole (DAPI), propidium iodide (PI), and MTT [3-(4, 5-Dimethylthiazol-2-yl)-2, 5-Diphenyltetrazolium Bromide] were obtained from Sigma-Aldrich, United States. The other required chemicals were procured from Hi-Media (Swastik Disha Business Park, *via* Vadhani Industrial Estate, L.B.S. Marg, Mumbai, Maharashtra) and Merck (Dr. Annie Basent Road, Worli, Mumbai Maharashtra) India. *Anabaena variabilis* was procured from the Indian Agriculture Research Institute (IARI) New Delhi and biofilm-forming strain *C. albicans* (MCC-1151) MCC stand for microbial culture collection was obtained from the National Centre for Microbial Resource (NCMR) Pune, India, respectively. The fungus was sub-cultured on yeast peptone dextrose (YPD) agar at 30°C for 24 h to obtain a fresh culture, which was used throughout the experiment. One colony was taken from the fresh primary cultures and re-suspended in (YPD) broth to attain a concentration of 1 × 10^6^ cells/mL.

### Synthesis and Characterization of Silver Nanoparticles

Bioreduction of silver nitrate (AgNO_3_) to AgNPs was done using *A. variabilis* cell extract. For AgNPs synthesis 90 ml AgNO_3_ (1mM) at 30°C and pH 7.4 was mixed in 10 mL cell extract under static condition for 24 h, and spectra were recorded at 300–700 using UV–Vis spectrophotometer (Labtronics, United States). AgNO_3_ without extract was used as a control. The morphological studies of the biogenic nanoparticles were studied by transmission electron microscope (TEM). The energy-dispersive X-ray (EDX) was performed using scanning electron microscopy (Abdallah and Ali) to find out the percentage elemental silver inside the synthesized nanoparticles. Additionally, FTIR measurements were performed on KBr pellet with diluted AgNPs for characterization of biomolecules inside the synthesized AgNPs and the cell extract. X-ray diffraction (XRD) studies of nanoparticles were performed for determining the nature and size of nanoparticles using the Debye–Scherrer equation. All the above characterization was done as detailed in our earlier publication (Ahamad et al., 2021).

### Effect of AgNPs on Growth Kinetics of Biofilm Forming *Candida albicans*

Growth kinetics assay of *C. albicans* was performed by modified protocol of Lee et al. (2019). In brief, an overnight culture of *C. albicans* was adjusted to 0.1 OD at 600 nm in fresh YPD broth media. Different concentrations 1/2 MIC, MIC, and 2MIC (6.25, 12.5, and 25 μg/mL) of AgNPs were added and incubated at 30°C in dark. Then; optical density was noted at 600 nm (OD_600_) for 24h using Labomed Spectrophotometer Labomed, Inc., S. La Cienega Blvd. Los Angeles, CA, United States.

### Effect of AgNPs on Morphology of Biofilm Forming *Candida albicans*

A slightly modified methodology of Lyu et al. (2016) was used to detect morphological alterations of fungal cells in the presence of AgNPs using scanning electron microscopy. The fungal cell suspension (1 × 10^6^ cells/mL) was exposed to MIC and 2MIC (12.5, 25 μg/mL) of AgNPs followed by incubation at 30°C for 3 h. The cells were centrifuged at 3000 revolutions per minute (RPM) three times for 5 min, and the resulting pellet was fixed for 3 h at 4°C with 2.5 percent (v/v) glutaraldehyde. The samples were fixed in 1 percent osmium tetroxide in PBS for 1 h at room temperature after three washes with 0.1 percent phosphate buffer saline (PBS). The fixed sample was cleansed in PBS before being dehydrated for 10 min with ethanol. The samples were dried with CO_2_ to a critical temperature, then coated with a thin layer (20–30 nm) of gold-palladium and examined with scanning electron microscope (Carl Zeiss Pvt. Ltd., UK Model ZEISS: Evo 18a) untreated *C. albicans* cell used as a control.

### Effect of AgNPs on Nuclear Condensation in Biofilm Forming *Candida albicans*

Nuclear condensation/fragmentation was performed by using a DNA-specific fluorescent dye 4-6-diamidino-2-phenylindole (DAPI). Healthy *C. albicans* were washed with PBS and incubated with AgNPs at 30°C 120 RPM for 4 h. After the incubation, cells were resuspended in PBS then centrifuge at 300 RPM for 5 min. In the next step, washed cells were treated with DAPI (1 μg/mL) and put at 30°C in dark. To examine nuclear staining, glass slides were prepared and observed under confocal laser scanning microscope (Leica) Wetzlar, Germany (Madeo et al., 1997).

### Effect of AgNPs on Membrane Permeabilization of Biofilm Forming *Candida albicans*

To determine the effect of AgNPs on membrane permeability, the propidium iodide (PI) uptake assay was used with minor modifications using flow cytometer (Seyedjavadi et al., 2020). *Candida albicans* cells were cultivated and diluted to 1 × 10^6^ cells/mL, and then incubated for 3 h at 28°C with consistent shaking at 120 RPM along with AgNPs of 1/2 MIC, MIC, and 2MIC (6.25, 12.5, and 25 μg/mL). After that, the filtered PI solution (50 μg/mL) was added and incubated for 15 min in the dark at 25°C before being washed with PBS. The percentage of PI-positive cells was determined using a Calibur Flow Cytometer with fluorescence-activated cell sorting (FACS) (BD Biosciences, San Jose, CA, United States). PI-treated and untreated fungal cells served as the negative control and blank, respectively.

### Fluorescence Microscopy

*Candida albicans* cells (1 × 10^6^ cells/mL) were treated with 1/2 MIC, MIC, and 2MIC (6.25, 12.5, and 25 μg/mL) of AgNPs before being incubated at 28°C for 3 h with continual shaking (120 RPM). The suspension was then rinsed in PBS and incubated with the PI solution (50 μg/mL) for 15 min at 25°C in the dark. Fluorescence microscopy (Eclipse 80) was used for the microscopic analysis at excitation/emission wavelength: 530/590 nm filter (Wang et al., 2015).

### Effect of AgNPs on Reactive Oxygen Species Induction in *Candida albicans*

The fluorescent probe 2′, 7′-dichlorofluorescin diacetate (DCFDA), a non-fluorescent chemical under normal conditions, was used to examine intracellular ROS produced in cells following AgNPs treatment. After internalization of DCFDA in the cells, it is hydrolyzed by cellular esterase to a non-fluorescent product, which is then oxidized by cellular ROS to a highly fluorescent 2′7′-dichlorofluorescein (DCF) compound. The cells were cultured overnight at 37°C, subsequently harvested by centrifugation (3500 RPM for 5 min) and treated for 24 h at 37°C with AgNPs 1/2 MIC, MIC, and 2MIC doses. Cells were harvested after 24 h by centrifugation at 3500 RPM for 5 min at 4°C, washed three times with PBS, and incubated for 30 min at 37°C in the dark with 1000 μL of 25 mM DCFH-DA. After that, the cells were harvested, and then washed in PBS, and flow cytometry (BD Biosciences, San Jose, CA, United States) was used to examine them. As a control, samples that had not been treated with AgNPs were used (Bezza et al., 2020).

### Effect of AgNPs on Fungal Cell Cycle

Log-phase cells of *C. albicans* (1 × 10^6^ cells) were collected and treated with AgNPs (at 20 times the MIC) in a YPD medium (Endo et al., 1997). The cells were washed with PBS and fixed with 70% ethanol overnight at 4°C after an 8-h incubation period. RNase-A was added to the cells at a concentration of 200 μg/mL, and the combination was allowed to react for 2 h at 37°C. For DNA staining 50μg/mL propidium iodide was added and incubated for 1 h at 4°C in the dark. A fluorescence-activated cell sorting (BD Biosciences, San Jose, CA, United States) FACS Calibur flow cytometer was used to perform the flow cytometric analyses.

### Determination of Hemolytic Activity of AgNPs

The hemolytic activity of AgNPs was assayed as described previously (Jadhav et al., 2018). In a brief, a healthy donor’s red blood cells (hRBCs) were collected in the presence of an anticoagulant and washed three times in PBS. Different concentrations 1/2 MIC, MIC, and 2MIC (6.25, 12.5, 25 μg/mL) of AgNPs were added to the suspension of RBCs (4%, v/v) in PBS to a final volume of 1 ml and incubated at 37°C for 35 min. Samples were then centrifuged for 2 min at 2000 RPM and the release of hemoglobin in supernatant was measured at 540 nm. For negative and positive controls, hRBCs in PBS (A blank) and in 1%Triton X-100 were used, respectively. The percentage of hemolysis was calculated as follows:


Hemolysis(%)=[(Atestsample-Ablank)/(ATriton-Ablank)]×100

### Effect of AgNPs on Cell Surface Hydrophobicity of Biofilm Forming *Candida albicans*

*Candida albicans* were grown in a YPD medium at 37°C for 48 h, treated with AgNPs 1/2MIC, MIC, and 2MIC (6.25, 12.5, and 25 μg/mL) doses, and kept at 37°C, for overnight in an orbital shaker, followed by 120 RPM. Cells were harvested by centrifugation at 5000 RPM for 5 min, followed by three PBS washes. The cells were resuspended in 5 ml of PBS at a concentration of 1 × 10^6^ cells per milliliter. Under vacuum, the cell suspension was filtered with a 0.22 m filter. To achieve constant moisture content, the membrane was dried for 35 h in a Petri plate containing 1 percent (w/v) agar in water containing 10% (v/v) glycerol. The percentage of hydrophobicity was calculated by the method of Silva-Dias et al. (2015).

### Effect of AgNPs on Yeast to Hyphal Transition

The dimorphic switching characteristics of *C. albicans* were performed using the previously described protocol (Saibabu et al., 2020). The overnight grown cultures were harvested and washed thrice with PBS, resuspended in PBS, and further incubated at 37°C in dark for 6 h to induce starvation. Then cells were transferred to the appropriate media, such as N-acetyl glucosamine (5 mM), for hyphal growth in the presence and absence of biogenic AgNPs, and hyphae were observed under the microscope at magnifications of 40x.

### Effect of AgNPs on Biofilm Formation and Disruption

*Candida* biofilms were checked on the polystyrene surface in 96-well plates as described previously (Saibabu et al., 2020). A cell suspension of 1 × 10^6^ cells/mL was prepared in PBS, and 100 μL was injected in each well after an overnight culture. To allow cells to attach to the surface, the plates were incubated for 90 min at 37°C and 50 RPM. The wells were gently washed two to three times with PBS to remove the non-adhered cells. The plates were loaded with 200 mL of YPD medium and 1/2 MIC, MIC, and 2MIC (6.25, 12.5, and 25 μg/mL) of AgNPs, and then incubated at 37°C for 24 h to see fungal biofilms growth. For biofilm disruption assays, 24 h grown biofilms were treated with 1/2 MIC, MIC, and 2MIC (6.25, 12.5, and 25 μg/mL) of AgNPs for 24 h. Wells were cleaned after incubation to remove any planktonic cells, and biofilms were measured using the MTT test.

### Effect of AgNPs on Qualitative Imaging of Biofilms Using Confocal Laser Scanning Microscopy

Biofilms were formed on glass-bottomed Petri dishes in the presence or absence (control) of AgNPs. Briefly, biofilms were treated with AgNPs 1/2 MIC, MIC, and 2MIC (6.25, 12.5, and 25 μg/mL). Samples were washed in PBS, dyed with Calcofluor white (sigma), which binds with the fungal cell wall chitin residues, and the morphologies of biofilms were imaged using an immersion lens (40x) for CLSM imaging (Leica) Wetzlar, Germany. Imaris redeveloped the recorded images and showed them as three-dimensional structures (Kagan et al., 2014).

### Statistical Analysis

All studies were done in triplicate. The findings were presented as mean ± standard deviation and analyzed using the Student *t*-test, with only *P* ≤ 0.05 being considered statistically significant.

## Results

For synthesis of AgNPs, cell extracts of *A. variabilis* (a cyanobacterium) were added into AgNO_3_ (1mM) in 1:9 ratios, subjected to continuous stirring at room temperature 28 ± 2°C up to 24 h. The color transformation from colorless to radish brown indicated synthesis of AgNPs and UV–visible spectrum was recorded from 300 to 700 nm **(S1)**. The size range of synthesized AgNPs was found 11–15 nm, with crystalline uniform spherical shape confirmed by TEM and XRD analysis.

Our earlier finding on biogenic AgNPs derived from *A. variabilis* showed antibacterial, antifungal, antioxidant (Ahamad et al., 2021). Higher inhibition of biofilm forming *C. albicans* by AgNPs prompted us to extend our finding on antifungal activity of AgNPs with special references to *C. albicans* biofilm. Throughout the study of AgNPs, MIC determined earlier as 12.5 μg/mL was used as a baseline for designing a new experiment. In the present investigation, besides AgNPs effect on biofilm formation and desorption, the mechanism of action of AgNPs was assessed with respect to growth inhibition, cell membrane permeability, reactive oxygen species, surface morphology, cell cycle analysis, DAPI staining, yeast to hyphae transformation, and cell surface hydrophobicity.

### Effect of AgNPs on Growth Inhibition Kinetic in *Candida albicans*

**FIGURE 1:**
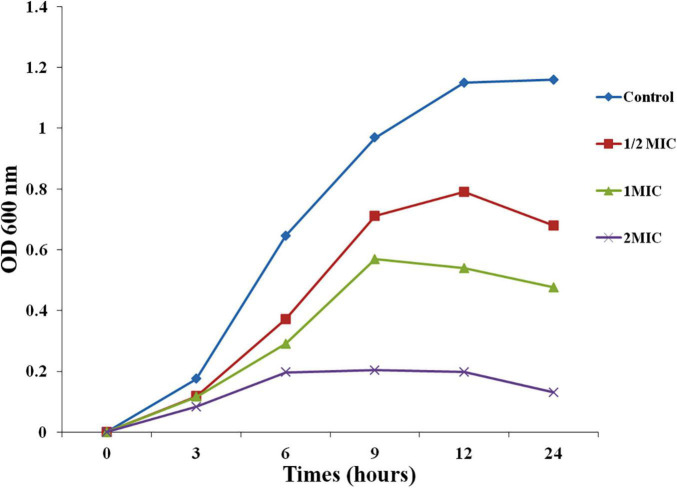
Effect of AgNPs on growth curve of *C. albicans.*

For growth inhibitions, kinetic of biofilm forming *C. albicans* was done at 1/2 MIC, MIC, and 2 MIC (6.25, 12.5, 25 μg/mL) of biogenic *A. variabilis* derived AgNPs. Growth inhibition was measured at 600 nm. With increasing MIC, the concentration of AgNPs growth of test organism decreases gradually in comparison to the control (Figure 1). Time taken for maximal growth in control was 12 h. The optical density of AgNPs increases at low concentrations but remains nearly constant at higher concentrations (MIC and 2MIC). AgNPs suppressed *C. albicans* development, based on the current findings.

### Effect of AgNPs on Cell Surface of *Candida albicans*

**FIGURE 2:**
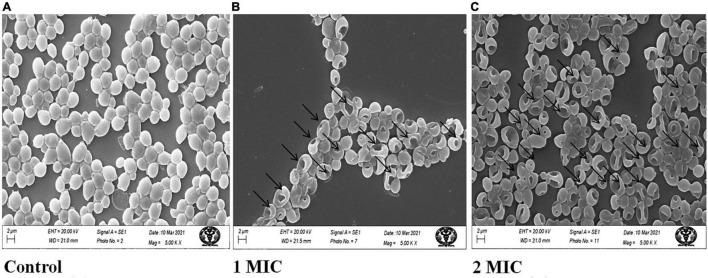
Scanning electron microscopic images of *C. albicans* treated with AgNPs. **(A)** Without AgNPs treated/control. **(B)** With 1MIC treatment. **(C)** With 2MIC treatment.

Scanning Electron Microscopy (SEM) images of the control and treated AgNPs on *C. albicans* showed that untreated cells retained normal form and smooth surfaces (Figure 2A), while AgNPs treated cells confirm deep wrinkles and deformity (arrows) after AgNPs treatment (Figures 2B,C).

### Effect of AgNPs on DNA Condensation in *Candida albicans*

**FIGURE 3:**
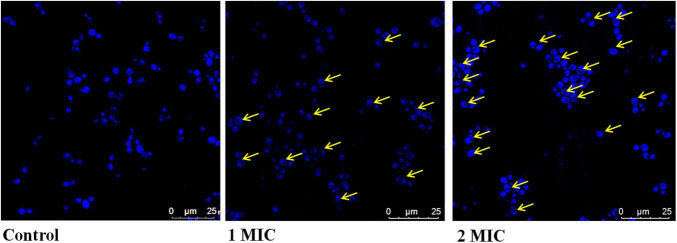
Effect of AgNPs on DAPI staining of *C. albicans.*

In the DNA condensation study, we assessed the effect of AgNPs on DNA integrity in *C. albicans*. Basically, cells with damaged/condensed DNA are unable to repair their DNA properly, causing cell death (Hong et al., 2004). In this study, DAPI was used to monitor nuclear condensation and fragmentation related with morphological changes in the nucleus. The minor groove of A-T rich regions of DNA is where DAPI binds (Daniel and DeCoster, 2004). In the present result of nuclear condensation, AgNPs treated *C. albicans* have nuclear abnormalities associated with oxidative stress and apoptosis than untreated cells (Figure 3).

### Effects of AgNPs on Cell Membrane Permeability of *Candida albicans*

**FIGURE 4:**
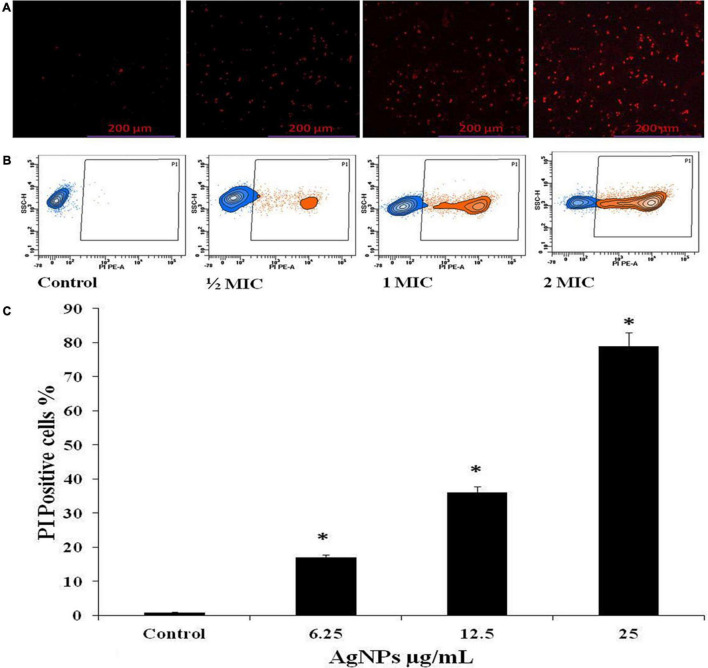
Effect of AgNPs on the cell membrane permeability of *C. albicans*, measured with propidium iodide (PI). **(A)** Fluorescence microscopy. **(B)** Flow cytometry. **(C)** Histogram analysis shows the percentage of PI-positive in *C. albicans* cells. * to represent the statistical significance which means *p*-value is less than 0.05.

The effect of AgNPs on the fungal cell membrane integrity was examined qualitatively by fluorescence and quantitatively by flow cytometric method using PI. Only damaged membranes allowed PI to bond with nucleic acids, resulting in a red glow under a fluorescence microscope. With increasing concentrations of AgNPs from 6.25, 12.5, and 25 μg/mL, the number of damaged dead *C. albicans* cells grew steadily; the control, on the other hand, had no fluorescence (Figure 4A). The same concentration of AgNPs was used in flow cytometry assay to quantify the PI uptake; by *C. albicans* and the percentage of PI uptake 17%, 36%, and 79% with 1/2 MIC (6.25), MIC (12.5), and 2MIC (25 μg/mL), AgNPs doses (Figures 4B,C). Membrane damage, such as the creation of holes, channels, carpets, or detergent activity, could cause enhanced membrane permeabilization.

### Effect of AgNPs on Reactive Oxygen Species Induction in *Candida albicans*

**FIGURE 5:**
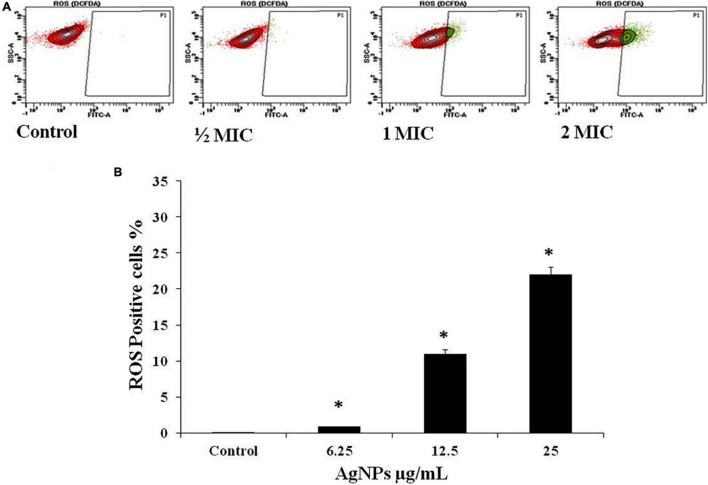
Effect of AgNPs on ROS generation in *C. albicans*, flow cytometry using DCFH-DA. **(A)** Cells were treated with 1/2 MIC (62.5 μg/mL), MIC (12.5 μg/mL), and 2MIC (25 μg/mL) for 3 h and stained with DCF-DA. **(B)** Histogram for percent ROS generation. * to represent the statistical significance which means *p*-value is less than 0.05.

The intracellular reactive oxygen species (ROS) production was evaluated on Flow Cytometer with DCFH-DA in *C. albicans* cells after treatment with AgNPs at 1/2 MIC (6.25 μg/mL), MIC (12.5 μg/mL), and 2MIC (25 μg/mL), for 3 h. A significant gradual increase in fluorescence was observed due to oxidation of DCFH-DA to DCF by intracellular ROS. The percentage of ROS-positive staining increase was 0.9%, 11%, and 22.2% with 1/2MIC, MIC, and 2MIC, respectively (Figures 5A,B).

### Effect of AgNPs in Cell Cycle of *Candida albicans*

**FIGURE 6:**
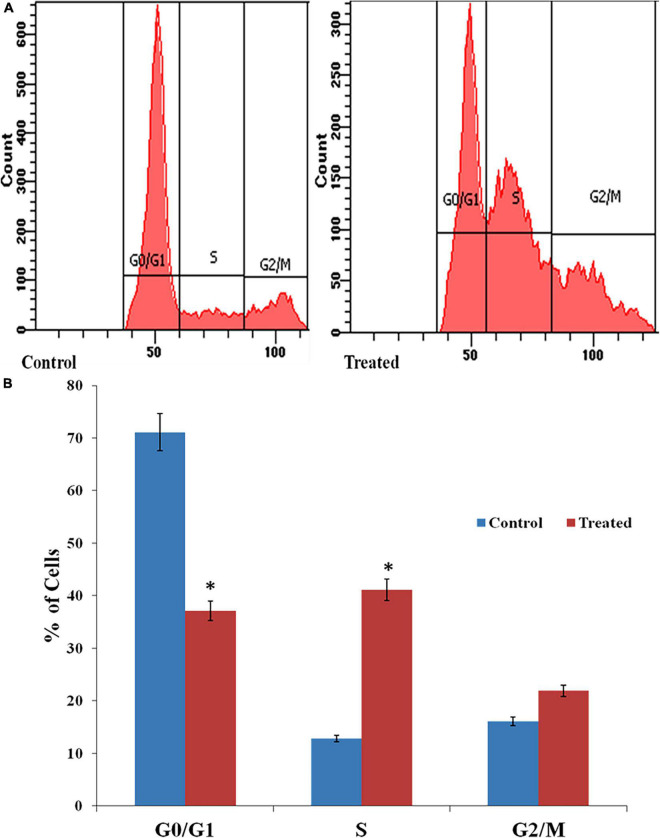
**(A)** Effects of AgNPs on the cell cycle of *C. albicans* detected through fluorescence-activated cell sorting (FACS). **(B)** Histogram of percentage of cells in different stages of the cell cycle. * to represent the statistical significance which means *p*-value is less than 0.05.

The DNA content of the AgNPs treated cells was assessed by flow cytometry after staining with propidium iodide (PI). PI is a DNA-staining dye that intercalates between the bases of DNA or RNA molecules (Ansari et al., 2016). AgNPs treated cells in the S phase were increased by 39.7% than control (drug free). The percentage of treated cells in the G2/M phase also increased by 5.8%, than control while, in the G1 phase, exhibited significantly decreased (about 36%) after treatment of AgNPs (Figures 6A,B).

### Effect of AgNPs in Hemolytic Activity

The toxicity of AgNPs was evaluated by hemolytic assay using the hRBCs which were isolated and subjected to AgNPs with increasing concentrations. In the present study, we observed only 4% hemolytic activity of AgNPs in comparison to the Triton-X used as a positive control which causes 100% hemolysis (**S2**).

### Effect of AgNPs on Hydrophobicity of Biofilm Forming *Candida albicans*

**FIGURE 7:**
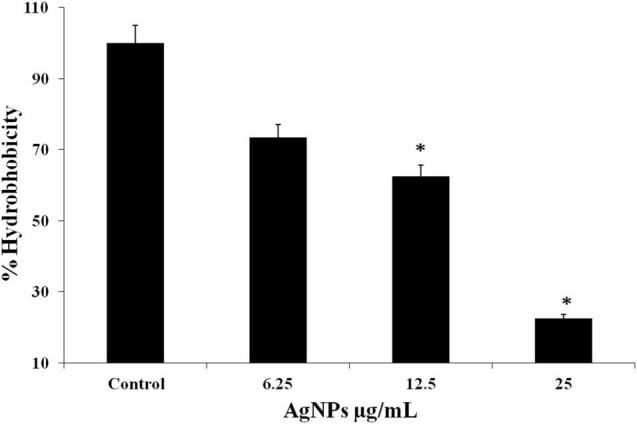
*C. albicans* hydrophobicity after treatment with AgNPs measured according to microbial adhesion assay to hydrocarbons (MATH) test. * to represent the statistical significance which means *p*-value is less than 0.05.

Hydrophobicity is the characteristic related with biofilm formation. *Candida albicans* displayed the good values of hydrophobicity (Figure 7). The percentages hydrophobicity of the cells was gradually decrease with increasing the concentration of AgNPs and was observed 22.5%, 62.5%, and 73.5% along with 1/2MIC, MIC, and 2MIC, respectively.

### Effect of AgNPs on Yeast-to-Hyphal Transition of *Candida albicans*

**FIGURE 8:**
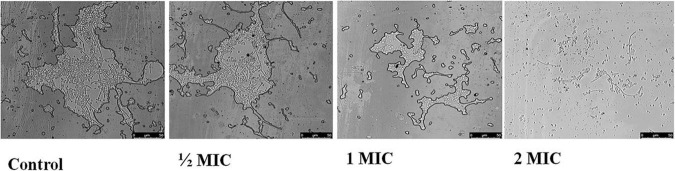
Effect of AgNPs on yeast to hyphal transition in the hyphae inducing media (N acetyl-glucosamine) (control) in *C. albicans* after 4 h at 37°C (magnification 40×).

In biofilm-forming *C. albicans*, the yeast to hyphal transition is the most important factor determining pathogenicity (Lara et al., 2015). The effect of AgNPs on this morphological switching condition was examined; cells were incubated at 37°C in N-acetylglucosamine (Glc-NAc) a nutrient-poor medium. In contrast to untreated *Candida* cells, which were able to express hyphal form, AgNPs treated cells almost completely lacked hyphae. It is suggested that AgNPs act as a potent inhibitor of the morphogenetic switching (Figure 8).

### Effect of AgNPs on Pre and Mature Biofilm

**FIGURE 9:**
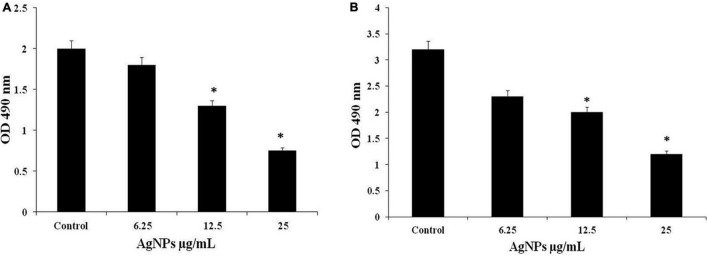
**(A)** Effect of AgNPs on *C. albicans* biofilm formation. **(B)** Biofilm disruption through MTT assay. * to represent the statistical significance which means *p*-value is less than 0.05.

AgNPs 1/2 MIC, MIC, and 2MIC treatment to *C. albicans* during MTT assay indicated gradual reduction in absorbance at 490 nm indicating that AgNPs inhibited formation of biofilm. The viability of biofilm was calculated in terms of percentage, and the inhibition in biofilm was observed 10%, 35%, and 62.5% along with the doses of 1/2 MIC, MIC, and 2MIC of AgNPs (Figure 9A). Treatment of the same doses of AgNPs on mature biofilm showed the gradual reduction in absorbance representing the disruption of mature biofilm. The viability of biofilm disruption was observed 28%, 37.5%, and 62.5% with 1/2 MIC, MIC, and 2MIC doses of AgNPs (Figure 9B). The present study result suggested that AgNPs are effective on biofilm formation as well as disruption.

### Qualitative Inhibition of Biofilms

**FIGURE 10:**
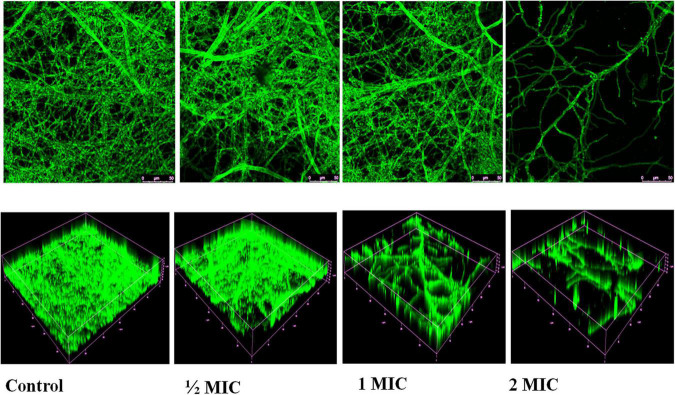
Confocal laser scanning microscopy images of biofilms untreated/control and treated with AgNPs. Volume representation of spatial distributions of biofilm stained with Calcofluor White at 40× magnification.

During qualitative degradation of biofilm in *C. albicans* was examined with Calcofluor white dye shows dense biofilm of *C. albicans* in untreated (control) cells while, treated with nanoparticles indicates the decrease the density of biofilm in concentration dependent manners. Using confocal laser scanning microscopy (CLSM), 2D and 3D images of the effect of AgNPs treatment on biofilm eradication were obtained depicted in Figure 10.

## Discussion

*Anabaena variabilis* is a prokaryotic photosynthetic filamentous cyanobacterium. Its short generation time and ability to withstand a wide spectrum of environmental stresses with low nutritional requirement costs make it a preferred candidate for nanoparticle synthesis. Cell-free extract of cyanobacteria contains sufficient protein which is capable of reducing metal ions into their insoluble form and then to nanocrystals. For AgNPs synthesis, 90 ml AgNO_3_ (1mM) at 30°C and pH 7.4 was mixed in 10 mL cell extract under static condition for 24 h, and a spectrum was recorded at 300–700. The color transformation from colorless to radish brown initially indicates synthesis of AgNPs. The size range of synthesized AgNPs was found 11–15 nm, with crystalline uniform spherical in shape confirmed by TEM and XRD analysis (Ahamad et al., 2021).

To investigate the possible mechanism of action of AgNPs, a growth inhibition experiment of *C. albicans* was carried out. AgNPs possessed antifungal potential against *C. albicans* cells, our findings, and the concentration of AgNPs was correlated to the antifungal activity. AgNPs at concentrations of 6.25, 12.5, and 25 μg/mL suppress the development of *C. albicans* cells throughout the incubation period. *Candida albicans* cell growth is considerably slowed at concentrations of 6.25 and 12.5 μg/mL for up to 9 h. The results with 1/2MIC and MIC were better in the next phase, which lasted from 9 to 24 h. Optimal density for the control and biogenic AgNPs during the first 3 h lag phase showed a slight variation range indicating acclimatization potential of *C. albicans*. The findings of the growth inhibition kinetics revealed that cell growth inhibition occurs quickly at 2MIC AgNPs dosages and is practically constant from 6 to 12 h, with a minor reduction in the graph at 24 h (Figure 1). Abdallah and Ali (2021) also reported antifungal activity of AgNPs synthesized by *Lotus lalambensis* Schweinf leaf extract.

In this study, *C. albicans* cells treated with AgNPs showed significant shrinkage and morphological changes, indicating increased membrane permeability (Figure 2). These findings, along with the results of membrane permeability tests, support membrane rupture, but the specific mechanism of AgNPs activity is unknown. However, a *C. albicans* SEM experiment was carried out to visualize the morphological alterations generated by AgNPs treatment in *C. albicans* cells. A similar result was also reported for chemically synthesized AgNPs (Radhakrishnan et al., 2018). In the present study, data revealed that AgNPs may induce fungal cell death either directly by interfering with cellular contents, such as nucleic acids and proteins.

In the present study, DAPI staining was performed; the results showed that enhanced DAPI staining in the presence of AgNPs revealed the damage of nucleus (Figure 3). AgNPs interact with DNA, causing nuclear condensation and DNA fragmentation, according to our findings. Nanoparticles that bind directly to DNA have the potential to cause cell division failure, which leads to apoptosis.

The antifungal efficacy of AgNPs is associated with membrane permeabilization. AgNPs could reach the cell membrane via passing the cell wall during the exponential growth phase of the fungal cell, since it has been proved that increasing porosity allows molecules with low molecular weight to pass through the fungal cell wall (Różalska et al., 2018). In addition, any alteration in the permeability of the fungal cell membrane could lead to the loss of internal components, particularly ions. The results of our PI uptake assay revealed that AgNPs can compromise the integrity of the fungal cell membrane and cause membrane permeabilization as the concentration of AgNPs rises. The present investigation of the PI uptake assay was observed with 6.25, 12.5, and 25 μg/mL of AgNPs; 17%, 36%, and 79% of cells were PI positive (Figure 4). According to Nisar et al. (2019), the enhanced permeability of *C. albicans* cellular membrane caused by AgNPs exposure could be due to contact with negatively charged components of the fungal surface (such as phosphatidyl serine and phosphatidyl inositol) that destabilize the membrane barrier. ROS are produced instantaneously during cellular metabolism and are important for signaling and homeostasis. Various environmental conditions cause an increase in the formation of ROS, which causes severe damage to cell structures. In addition to increasing cell membrane permeability, high ROS destroys a number of substances, including nucleic acids, proteins, and lipids that lead to cell death (Sharma et al., 2009; Jalal et al., 2016, 2019).

Reactive oxygen species (ROS)-positive staining was identified in 0.9 percent, 11 percent, and 22.2 percent with 1/2 MIC, MIC, and 2MIC, respectively, in the current investigation (Figure 5). Based on this result as shown in Figure 5 we conclude that AgNPs cause ROS production in *C. albicans* cells, which causes lipid peroxidation of the cell membrane, which damages phospholipids directly and can also act as a cell death signal, leading programmed cell death, such type of statement reported previously (Seong and Lee, 2018). Based on previous study (Vazquez-Muñoz et al., 2019), it is known that enhanced ROS generation by the treatment of AgNPs leads to oxidative stress and consequently, denature of biomolecules and cell damage.

A flow cytometric analysis of the cell cycle was carried out to understand the physiological changes generated by AgNPs in fungal cells. In the present study, AgNPs halted the cell cycle in *C. albicans* at the S and G2/M phases (Figure 6). The SWE1p gene is believed to be a Wee1-family kinase that is expressed during late G1 and S phase and stops the cell from entering both mitotic and isotropic shifts. Since, SWE1p stabilization leads to the expression of cell cycle S phase inhibitory proteins so AgNPs induced cell cycle disruption could suggest that S phase associated genes like SWE1p might be up-regulated by AgNPs. Such type of cell cycle result was previously reported with perillyl alcohol (PA), a natural monoterpene alcohol by Ansari et al. (2016).

The hemolytic finding validates low toxicity of AgNPs with the development of AgNPs-based medicine; the question of their possible toxicity has recently gained attention. In this context, it is highly required to study the toxicity of nanomaterials on blood, specifically erythrocytes. Blood biocompatibility of nanoparticles is studied by evaluating the potential percent hemolysis when exposed to hRBCs, as nanoparticles are known to cause membrane damage and cell death (Carlson et al., 2008). The results of hemolysis assay suggest that the green synthesized AgNPs at <5% hemolysis, which lies within the biocompatible range in accordance to ISO/TR 7406 [the critical safe biomaterials hemolytic ratio (5%)] (Li et al., 2012).

*Candida albicans* has the ability to develop biofilms that make stronger their adherence to surfaces and increase their resistance to antifungal medicines (Forsberg et al., 2019). Although the exact processes that increase their resistance are unknown, some unfavorable factors are known to support *C. albicans* tolerate hard environments, such as matrix polysaccharide protection (Dominguez et al., 2019) as well as efflux pump overexpression (Kean et al., 2018). As a result, biofilm formation is currently a concern to both individual patients and healthcare facilities (Sears and Schwartz, 2017).

Biofilm formation is important for stable colonization in host tissues as well as resistance to environmental stresses like antifungal drugs and oxidative stress (Douglas, 2003; Seneviratne et al., 2008). It is well known that once a biofilm is formed, candidal cells within these biofilm become intrinsically resistant to all fungicides as well as physical and chemical sanitizing methods (Ramage et al., 2009; Sherry et al., 2017). Therefore, novel anticandidal and antibiofilm agents are urgently needed to treat these uncontrolled diseases.

Cell surface hydrophobicity (CSH) is usually considered a good indicator of adhesion ability of biofilm forming organism, i.e., substances with potential decrease hydrophobicity will inhibit biofilm formation (Panagoda et al., 2001). In the present study, AgNPs treated cells showed the percentages of cell surface hydrophobicity were 22.5%, 62.5%, and 73.5% along with 1/2MIC, MIC, and 2MIC, respectively (Figure 7). Gupta and Chhibber (2019) also reported hydrophobicity plummeted sharply in AgNPs treated cells compared to untreated cells. In the present study, result showed reduction in the hydrophobicity in a concentration-dependent manner.

Yeast to hyphae transition is an important virulence feature of *C. albicans* which helps invasion and systemic feature of the host tissues (Saibabu et al., 2020). The developing yeast to hyphal transition is an intriguing feature of *C. albicans* that facilitates adhesion and leads to biofilm formations, which are required for initiation of candidal pathogenesis (Harper, 2002; Modrzewska and Kurnatowski, 2015). The infection would be stopped if the transformation from yeast to hyphal form was hampered. Another study found that HIR genes (regulatory genes) are responsible for the yeast to hyphae transition and if the transfer from yeast to hyphal form is delayed, the infection will be stopped. HIR genes (regulatory genes) are responsible for the yeast to hyphae transition, according to another study, if this is not expressed then unable to convert from yeast hyphal form (Jenull et al., 2017). In the present study, biogenic AgNPs showed effective potential against the transition switching from yeast to hyphae (Figure 8). In the presence of AgNPs yeast to hyphae transition genes (HIR genes) might be suppressed lead to down-regulated. AgNPs might be targeted regulatory networks like environmental sensing, signaling, transcriptional modulators as well as chromatin modifications those are playing important role in the yeast to hyphae transition pathway which is pivotal for biofilms and virulence form (Maubon et al., 2014; Rajendran et al., 2016).

In the present study, AgNPs not only inhibited the formation of biofilm but also led to disruption of mature biofilms by *C. albicans* shown in Figures 9A,B, and confocal image of two-dimensional and three-dimensional biofilm disruption is depicted in Figure 10. The exact mechanism by which AgNPs suppress biofilm development is unknown. Różalska et al. (2018) proposed that antibiofilm activity was due to the AgNPs highly facile binding and increased penetration into the biofilm structure, disrupting the lipidome of cell membranes. Another plausible reason for AgNPs antibiofilm effectiveness is that they impede yeast morphogenesis; the inhibition of blastospores and hyphae formed by AgNPs also prevents Candida biofilm formation (Różalska et al., 2018). According to Lara et al. (2015), AgNPs antibiofilm action is mostly due to the rupture of the cell wall, which allows both yeast and filamentous *Candida* spp. to survive. The present study demonstrates AgNPs revealed a potential activity against the mature and preformed biofilms of *C. albicans.* Moreover, fluorescence, membrane permeability, SEM, DAPI and cell cycle result exhibit the potent antifungal activity against the *C. albican*s. Due to this reason, AgNPs may be utilized in the targeting of the virulence factor in *C. albicans* for developing a novel concept of drugs against candidiasis.

## Conclusion

The finding of the present investigation suggested that cyanobacterium (*A. variabilis*) derived AgNPs were effective against *C. albicans* via a combined mechanism of action involving cell membrane permeability, induction of ROS, and cell cycle study. The biofilm results like cell surface hydrophobicity, yeast to hyphal transition inhibition, eradicating established mature biofilms, and inhibiting biofilm formation revealed that AgNPs coating on medical devices considered a promising therapeutic tool against clinical life-threatening candidiasis. However, *in vivo* investigations of AgNPs are required before being employed in biomedical applications.

## Data Availability Statement

The original contributions presented in the study are included in the article/Supplementary Material; further inquiries can be directed to the corresponding author.

## Author Contributions

TF conceived and designed the experiments and supervised the present investigation. IA performed the experiments and prepared the draft. IA, FB, RA, and TF contributed to data analysis. IA, RA, and FB contributed to manuscript writing. PS and RK edited the manuscript. All authors have read and approved the manuscript.

## Conflict of Interest

The authors declare that the research was conducted in the absence of any commercial or financial relationships that could be construed as a potential conflict of interest.

## Publisher’s Note

All claims expressed in this article are solely those of the authors and do not necessarily represent those of their affiliated organizations, or those of the publisher, the editors and the reviewers. Any product that may be evaluated in this article, or claim that may be made by its manufacturer, is not guaranteed or endorsed by the publisher.
