# Supplementary material for: Antibiofilm Activities of Biogenic Silver Nanoparticles Against Candida albicans
Source: Front Microbiol. 2022 Jan 7;12:741493. doi: 10.3389/fmicb.2021.741493 (PMC8782275; doi:10.3389/fmicb.2021.741493)
Supplement: Supplementary file 1 [file Data_Sheet_1.docx]

Supplementary Material

**Antibiofilm activities of biogenic silver nanoparticles against *Candida albicans***

***Irshad Ahamad^a^, Fareha Bano^b^, ^c^Razique Anwer,* Pooja Srivastava^d^, Raj Kumar^d^, *Tasneem Fatma^a^****

^a^Cyanobacterial Biotechnology Lab, Department of Biosciences, Jamia Millia Islamia, New Delhi-110025, India

^b^Department of Biology, Faculty of Science and Arts Taibah University (Female Branch) Al Ula Campus City: Al Ula, Province: Al Madinah Al Munawarah Kingdom of Saudi Arabia

^c^Department of Pathology, College of Medicine, Imam Mohammad Ibn Saud Islamic University (IMSIU), Riyadh, Saudi Arabia

^d^Institute of Nuclear Medicine & Allied Sciences, Defence Research & Development Organisation (DRDO), Government of India, New Delhi-110054, India

*Email: [fatma_cbl@yahoo.com](mailto:fatma_cbl@yahoo.com)

**Supplementary Figure S1**- UV- visible spectrum of *A. variabilis* derived AgNPs

**
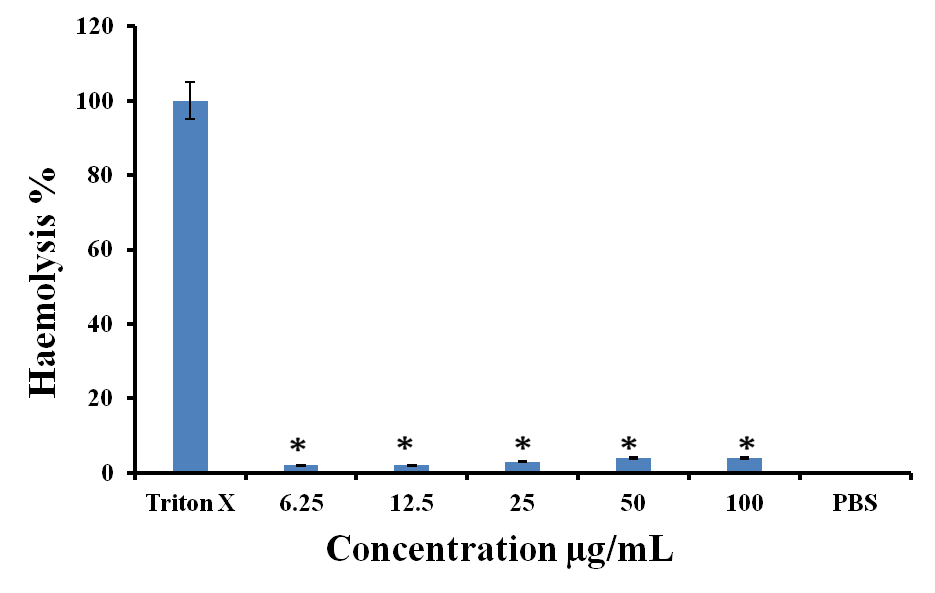
**

**Supplementary Figure S2**- Haemolytic activity of *A. variabilis* derived AgNPs
